# Development of a mammalian neurosensory full‐thickness skin equivalent and its application to screen sensitizing stimuli

**DOI:** 10.1002/btm2.10484

**Published:** 2023-01-03

**Authors:** Matthew Freer, Nicole Darling, Kirsty Goncalves, Kevin J. Mills, Stefan Przyborski

**Affiliations:** ^1^ Department of Biosciences Durham University Durham UK; ^2^ Procter & Gamble Cincinnati Ohio USA; ^3^ Reprocell Europe Ltd Glasgow UK

**Keywords:** capsaicin, inflammatory cytokines, neuroinflammation, neurosensitization, neurosensorial, skin model, substance P

## Abstract

Human skin equivalents (HSEs) are an increasingly popular research tool due to limitations associated with animal testing for dermatological research. They recapitulate many aspects of skin structure and function, however, many only contain two basic cell types to model dermal and epidermal compartments, which limits their application. We describe advances in the field skin tissue modeling to produce a construct containing sensory‐like neurons that is responsive to known noxious stimuli. Through incorporation of mammalian sensory‐like neurons, we were able to recapitulate aspects of the neuroinflammatory response including secretion of substance P and a range of pro‐inflammatory cytokines in response to a well‐characterized neurosensitizing agent: capsaicin. We observed that neuronal cell bodies reside in the upper dermal compartment with neurites extending toward the keratinocytes of the *stratum basale* where they exist in close proximity to one another. These data suggest that we are able to model aspects of the neuroinflammatory response that occurs during exposure to dermatological stimuli including therapeutics and cosmetics. We propose that this skin construct can be considered a platform technology with a wide range of applications including screening of actives, therapeutics, modeling of inflammatory skin diseases, and fundamental approaches to probe underlying cell and molecular mechanisms.

## 1 INTRODUCTION

Human skin equivalents (HSEs) are bioengineered 3D tissues that recapitulate aspects of skin structure and function in vitro. The use of HSEs has become increasingly popular as restrictions limit animal testing,^1^ and many applications of HSEs have been demonstrated including cosmetic science,^2^, ^3^, ^4^, ^5^, ^6^ disease modeling,^7^, ^8^, ^9^, ^10^, ^11^, ^12^ fundamental investigations,^13^, ^14^, ^15^, ^16^ impact of exposome on skin health,^17^, ^18^, ^19^, ^20^, ^21^, ^22^ hormonal influence^23^, ^24^, ^25^ and aging processes.^26^ Many basic HSEs contain only two primary cell types: keratinocytes that form a stratified epidermis and fibroblasts used to model a supporting dermal compartment.^5^, ^17^, ^27^, ^28^, ^29^, ^30^ This significantly limits the accuracy of predictive outcomes from HSE use, as the skin in vivo is a complex organ consisting of supporting cell types and appendages.

Skin neurosensitization is a response characterized by pruritis, erythema, and localized pain. It may arise due to an adverse reaction following topical application of a cosmetic or medication,^31^, ^32^ or an underlying chronic inflammatory skin condition such as atopic dermatitis.^33^, ^34^, ^35^ In vivo this response is partly mediated by cutaneous sensory neurons, the cell bodies of which are located within the dorsal root ganglion (DRG) and nerve fibers extend into the dermis and reside in close contact with cells of the epidermis.^36^, ^37^, ^38^ Sensory neurons are in‐part responsible for a cascade of events including the release of neurosensitizing, pro‐inflammatory and vasoactive factors that induce an unpleasant skin response that can impact the quality of life.^39^, ^40^

Understanding the molecular events involved in cutaneous neurosensitization is important not only for the safe and effective screening of cosmetics and therapeutics but also to provide insight into the pathogenic mechanisms of pro‐inflammatory skin disorders. However, for this pursuit, a suitable in vitro alternative to human skin is lacking due to most well‐characterized HSEs containing only basic cell types: keratinocytes and fibroblasts, modeling the epidermal and dermal compartments, respectively.^38^, ^39^, ^40^, ^41^, ^42^, ^43^, ^44^ A limited number of innervated, neuron‐containing HSEs have been described to date but either contain a poorly stratified epidermis,^45^ model epidermal interactions only^46^ or utilize exogenous animal‐derived extracellular matrix (ECM) constituents^47^ that inaccurately represent the biochemical and biophysical qualities of native human skin.

Previously we have described the development of a novel, robust, full‐thickness HSE that accurately recapitulates many facets of native human skin.^27^ This tissue construct is engineered using a porous, polystyrene scaffold populated by human dermal fibroblasts that secrete endogenous ECM, producing a robust dermal foundation upon which a stratified epidermis is constructed. This bioengineered HSE provides an in vitro alternative to human skin that accurately models structural and functional aspects of human skin including ECM architecture, barrier function, and epidermal organization.

In this study, we describe advances and modifications to our original HSE platform, whereby the inclusion of neurons produced an innervated tissue, capable of stimulation and neuropeptide release. We demonstrate the anatomically correct placement of extending neurites, in close contact with keratinocytes of the epidermis and the formation of a stratified and well‐organized epidermis upon an innervated dermal compartment. Most importantly, we demonstrate the release of neuropeptides and pro‐inflammatory cytokines following stimulation with a well‐characterized neurosensitizaing agent, capsaicin, known to induce an hyperalgesic response in vivo.^48^, ^49^, ^50^, ^51^

This demonstrates not only the development of a neuron‐containing full‐thickness HSE (FT‐HSE), of which few are described in the literature, but also the functionalization and expected response upon stimulation, providing a platform technology suitable for a wide range of applications. This novel, bioengineered construct can be applied to a comprehensive range of industrial and academic pursuits in vitro including topical cosmetic and therapeutic screening, fundamental approaches to gain insight into mechanisms involved in chronic inflammatory skin diseases, and the algesic response to painful stimuli.

## 2 RESULTS

### 2.1 Generation of sensory phenotype neurons

**FIGURE 1:**
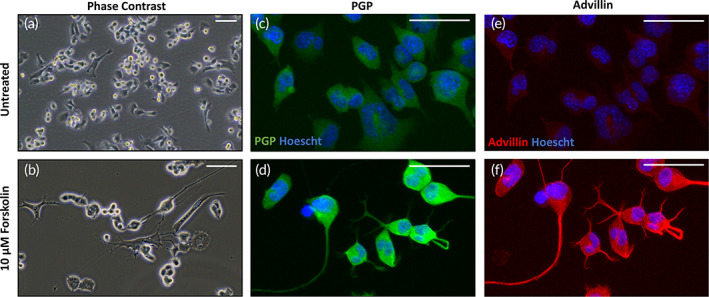
Forskolin differentiated F11 cells display properties of sensory neurons. F11 cells were cultured in 2D without forskolin (a,c,e) or treated for 24 h with 10 μM forskolin (b,d,f), a potent stimulus of neuronal differentiation. Phase‐contrast analysis reveals morphological changes induced through differentiation, including elongation of cells and the extension of neurites from individual perikarya. Immunofluorescence staining for neuronal marker PGP (c,d) and sensory neuron‐specific advillin (e,f) reveal an increase in expression of both markers following differentiation with neurite extension evident. PGP: green, advillin: red, Hoescht: blue. Scale bar: 50 μm

The F11 hybrid neuronal cell line is a commonly used model cell line and a fusion product of mouse neuroblastoma cells with embryonic rat dorsal‐root ganglion (DRG) cells. These well‐characterized cells are often used to generate a population of sensory‐like, functional neurons for in vitro studies of cutaneous sensitization and peripheral nervous system regeneration.^52^, ^53^, ^54^, ^55^ The morphology of undifferentiated cells appears heterogeneous from phase‐contrast micrographs (Figure 1a) and immunofluorescence reveals expression of PGP (Protein Gene Product 9.5) (Figure 1c) and advillin is low (Figure 1e). Both PGP 9.5 and advillin are biomarkers associated with a sensory neuron phenotype.^56^, ^57^

Following 24 h differentiation with 10 μM forskolin, a potent morphogen and inducer of neuronal differentiation,^55^, ^58^, ^59^ the morphology of cells appeared more dendritic as visualized through phase‐contrast microscopy (Figure 1b) with observable neurite outgrowth. Immunofluorescence analysis revealed an increase in expression of both PGP 9.5 (Figure 1d) and advillin (Figure 1f) with notable morphological differences compared with undifferentiated cells, including the extension of neurites from individual perikarya. These data suggest that forskolin induction results in the generation of a peripheral, sensory‐like subclass of neuron from the F11 cell line, suitable for incorporation into the HSE to produce a functional tissue, although not species matched.

### 2.2 Inclusion of sensory‐like neurons into dermal compartment

**FIGURE 2:**
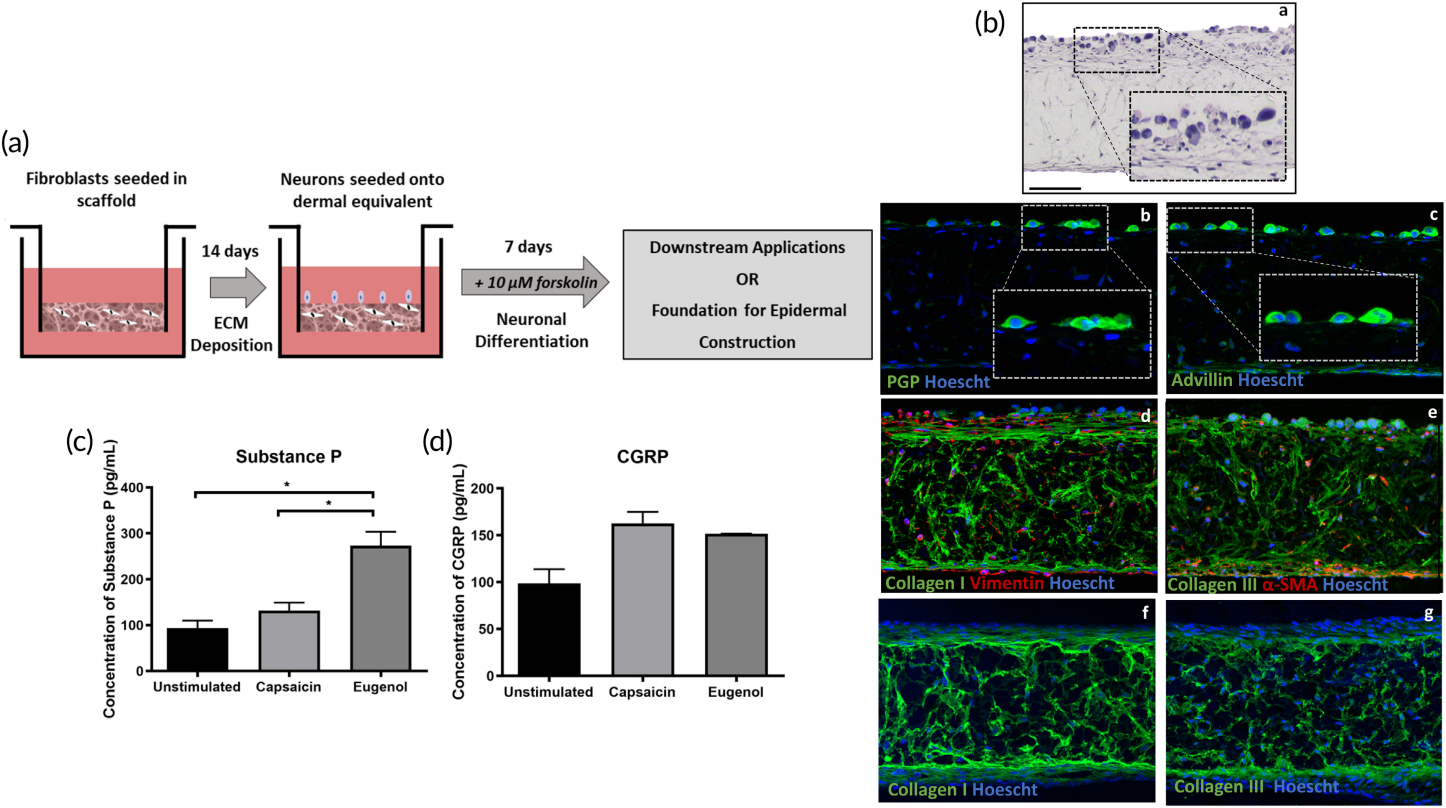
Addition of sensory neurons to dermal compartment to engineer a functional tissue. Dermal fibroblasts were allowed to populate the 3D scaffold and secrete endogenous extracellular matrix (ECM) for 14 days prior to addition of sensory neurons (a). Hematoxylin and eosin (H&E) staining (ba) reveals a fibroblast‐rich dermal compartment supported by the 3D scaffold, upon which neuronal cell bodies can be identified. The presence of neuronal cells on the apical surface of the dermis was confirmed through immunofluorescence staining for neural markers PGP (bb, green) and advillin (bc, green). Dermal composition remains unaffected by neuronal co‐culture with abundant immunofluorescence staining for ECM and fibroblast biomarkers Collagen I (green) and Vimentin (bd, red) and Collagen III (green) and α‐SMA (be, red) in the presence or absence (bf,g) of neurons. Immunofluorescence images are counterstained with Hoescht, which stains nuclei blue. Release of neuropeptides substance P (c) and calcitonin gene‐related peptide (CGRP) (d) is greatly increased following stimulation with known sensitizing agents: Capsaicin and eugenol as measured by ELISA. Data represent mean ± SEM, *n* = 3, *p < 0.05. Scale bar: 100 μm

Previously we have described the culture of neonatal human dermal fibroblasts within a porous polystyrene scaffold, and the subsequent secretion of an endogenous ECM that recapitulates many of aspects of native human dermis.^27^ Here, we have adapted this methodology to incorporate the inclusion of F11‐derived sensory‐like neurons into the dermal compartment (Figure 2a). Fibroblasts were seeded onto the porous scaffold and allowed to populate the scaffold and secrete their endogenous ECM proteins over a 14‐day culture period. Following dermal establishment, undifferentiated F11 cells were seeded onto the dermal equivalent and cultured for 7 days in the presence of 10 μM forskolin to promote neuronal differentiation and adoption of a sensory‐like phenotype. Following the establishment of a neural population, dermal compartments were either harvested for analysis or formed the foundation for FT‐HSE construction.

Neuronal cell bodies were visible, residing on the surface of the fibroblast populated scaffold as visualized through H&E staining (Figure 2ba). Positive immunoreactivity for both PGP 9.5 (Figure 2bb) and advillin (Figure 2bc) revealed that neurons have adopted a sensory‐like phenotype, consistent with forskolin induction in 2D culture. Furthermore, they do in‐fact reside on the surface of the dermal compartment, with no evidence of neuronal cell invasion into the scaffold itself, an important feature for subsequent epidermal construction.

Expression of ECM proteins Collagen I (Figure 2bd) and Collagen III (Figure 2be) was conserved in the presence of neurons with immunofluorescence staining revealing a dense network of both collagens within the scaffold. This dense ECM network of collagens remained unaffected in the presence of neurons and forskolin treatment when compared with age‐matched dermal compartments that lacked neurons (Figure 2bf,g). These proteins are secreted from the dermal fibroblasts, providing biochemical and biophysical support to the engineered tissue reflective of the native dermal microenvironment. Immunofluorescence staining reveals cells rich in vimentin (Figure 2bd), a pan‐fibroblast biomarker, and αSMA (Figure 2be), a mature myofibroblast biomarker, are visible within both the scaffold itself and lining the surface of the dermal compartment. This suggests that co‐culture of neuronal cells and fibroblasts has not had any negative effects on fibroblast viability or function, thus producing an intact dermal compartment with apical sensory‐like neurons.

These data demonstrate the localization of sensory‐like neurons upon an ECM‐rich dermis, however to further characterize this novel dermal system and demonstrate neuronal functionality, we measured the secretion of neuropeptides substance P (Figure 2c) and calcitonin gene‐related peptide (CGRP, Figure 2d). Substance P is released by nociceptive sensory neurons upon induction with a noxious peripheral stimulus.^60^ Similarly, CGRP is secreted by peripheral neurons and is a potent vasodilator, transmitter of nociception, and potentially a conductor of noxious stimulation.^61^ Due to these actions, we measured both release of substance P and CGRP upon stimulation with capsaicin and eugenol, both of which are well‐characterized stimulants of nociception and inflammation in the peripheral nervous system.^62^, ^63^

The concentration of substance P in the culture medium of innervated dermal equivalents was slightly increased upon stimulation with capsaicin and significantly increased following eugenol stimulation. Likewise CGRP concentration within the culture medium was greatly increased following capsaicin and eugenol stimulation. These data suggest that not only do the neurons possess the expected biomarkers and are visible on the surface of the dermal compartment, but that they behave in an anticipated manner through secretion of physiologically relevant neuropeptides upon stimulation. This demonstrates that the neurons are both viable and functional, providing a robust foundation upon which a stratified epidermis can be constructed.

### 2.3 Construction of a functional neurosensory full‐thickness skin equivalent

**FIGURE 3:**
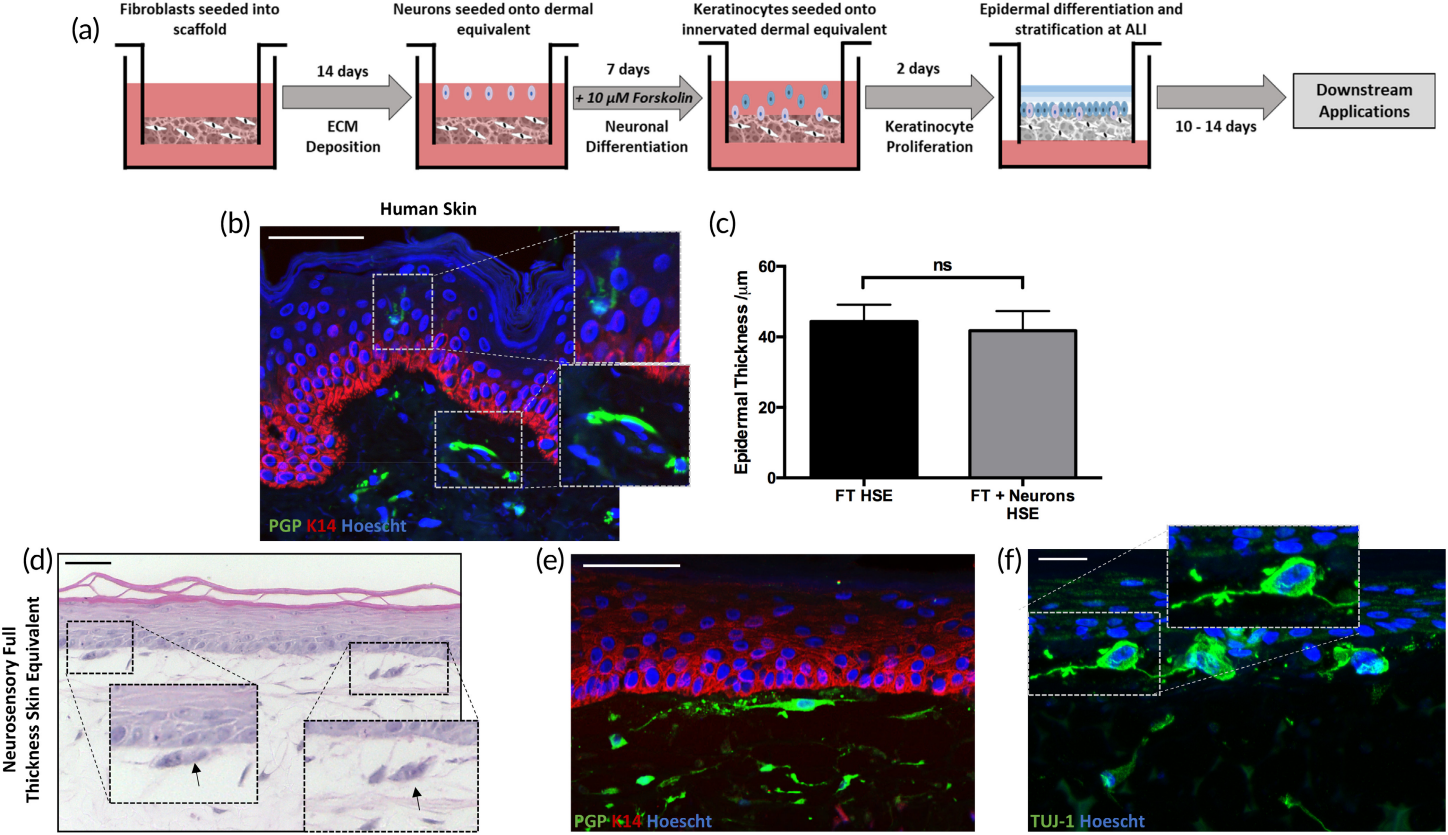
Incorporation of sensory neurons into full‐thickness skin equivalent supports epidermal anatomy. Neurons were allowed to proliferate on the surface of a dermal for 7 days prior to seeding of keratinocytes and formation of an epidermal compartment (a). Immunofluorescence staining of human skin (b) for neuronal biomarker PGP (green) and keratinocyte biomarker keratin‐14 (K14, red) reveals neuronal cell bodies located within the dermis, with neurites extending to the epidermis, in close proximity with keratinocytes. Epidermal thickness is unaffected by presence of sensory neurons within FT‐HSE (c). Data represent mean ± SEM, *n* = 9. Sensory neurons were successfully incorporated into FT‐HSE with perikarya (arrows) visible directly beneath epidermis as visualized by H&E staining (d). Immunofluorescence staining for PGP (green) and K14 (red, e) and pan‐neuronal marker TUJ‐1 (f) reveals neurons residing directly below the epidermis with neurites extending and in contact with keratinocytes. Hoescht counterstains nuclei blue in immunofluorescence images. Scale bars: 50 μm

In order to bioengineer an innervated FT‐HSE, we first created functional neuron‐containing dermal compartments upon which a population of neonatal keratinocytes was seeded in submerged culture for 2 days, to induce proliferation and for a further 10–14 days at the air–liquid interface (ALI) to promote stratification and keratinocyte differentiation (Figure 3a). This resulted in the formation of a FT‐HSE with a well‐organized epidermis upon a fibroblast and ECM‐rich dermal compartment, as visualized through H&E staining (Figure 3d). Neuronal cell bodies can be identified in the upper dermis, below the epidermis and in close contact with basal keratinocytes.

Immunofluorescence staining for the sensory neuron biomarker PGP 9.5 and basal keratinocyte biomarker keratin‐14 (K14; Figure 3e) reveals the location of sensory‐like neurons immediately below the epidermis with neuritic extensions in close proximity to basal keratinocytes. In native human skin (Figure 3b), PGP‐positive neurons can be identified within the dermis with free nerve endings extending up into the epidermis, interacting with keratinocytes. Although neurites do not visibly cross the basement membrane in our innervated HSE system, the close proximity or neurites to basal keratinocytes does suggest a degree of interaction. The location of neurons within the engineered FT‐HSE tissue is also demonstrated through positive staining for the pan‐neuronal marker TUJ‐1 (Figure 3f), which reveals neuronal perikarya positioned immediately below the epidermis with the extension of long neurites in close contact with basal keratinocytes.

We also demonstrate that the incorporation of neurons into the FT‐HSE had no negative effects on epidermal formation, structure, or thickness. A successful, well‐organized, stratified epidermis was adequately constructed onto a dermal compartment layered with sensory‐like neurons and further analysis revealed no significant difference in epidermal thickness (Figure 3c) between FT‐HSE containing neurons and those that did not.

**FIGURE 4:**
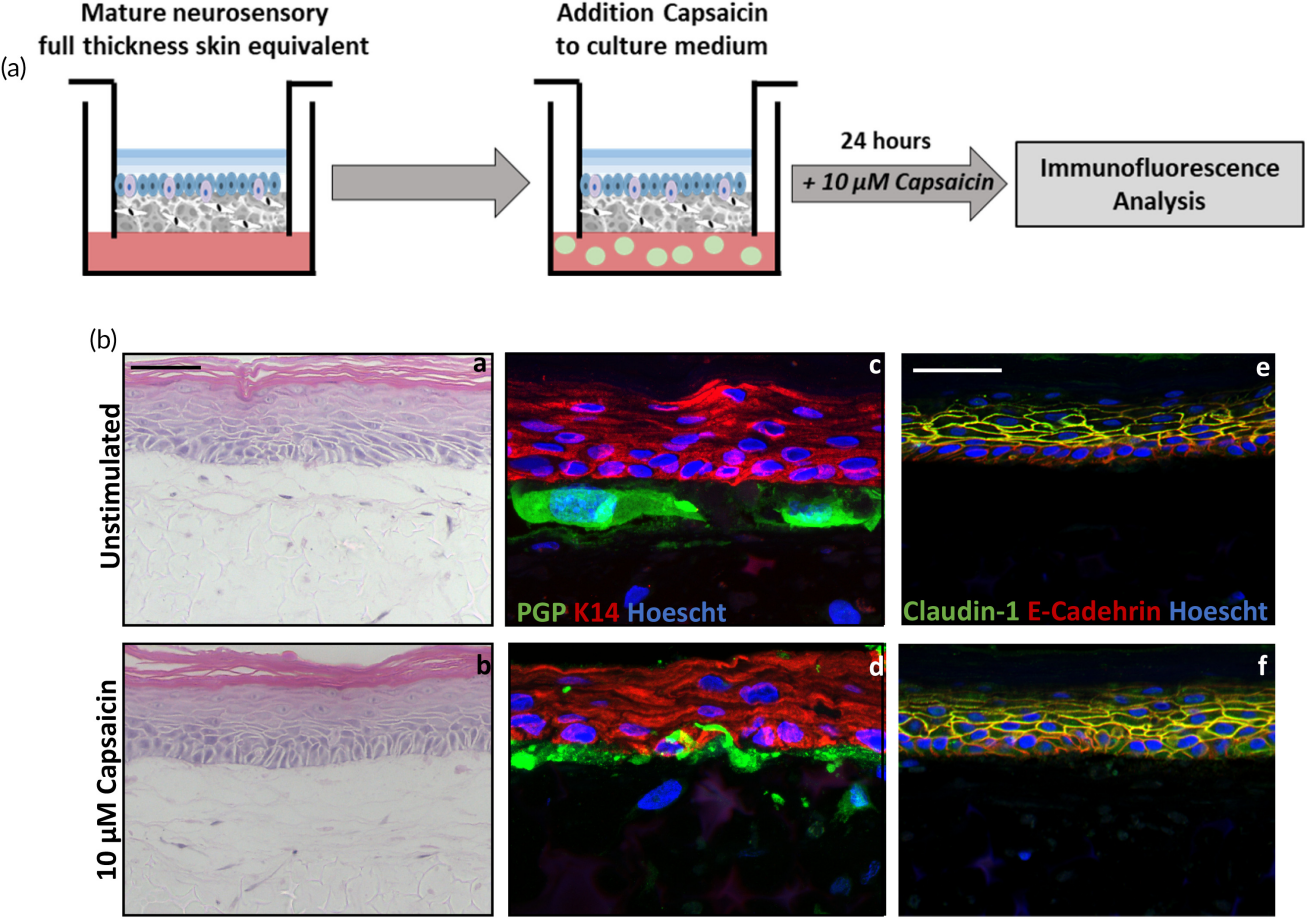
Capsaicin stimulation has no impact on skin equivalent structure. Neurosensory FT‐HSEs were exposed to 10 μM capsaicin for 24 h in their culture medium (a). Stimulation resulted in no significant alteration in epidermal structure as visible by H&E staining (ba,b). Immunofluorescence analysis (bc,d) reveals close contact between neurons (PGP, green) and keratinocytes (K14, red). Expression of junctional proteins (be,f) claudin‐1 (green) and E‐cadherin (red) appears unchanged by stimulation with capsaicin. Immunofluorescence images are counterstained with Hoescht which dyes nuclei blue.

These data demonstrate the expected tissue anatomy that compares well with in vivo skin, in that neurite extensions reside in close proximity to basal keratinocytes of the epidermis. However, in order to test whether neurons and keratinocytes indeed interact with one another and produce a functional tissue model, we examined the response of the innervated FT‐HSE to a known irritant and noxious agent: capsaicin, a well‐characterized stimulus of nociceptive peripheral neurons. Fully matured neuron‐containing FT‐HSEs at 14 days ALI were exposed to 10 μM capsaicin through addition to their culture medium for 24 h then subsequently harvested for analysis (Figure 4a).

Histological analysis (Figure 4ba,b) revealed epidermal structure remained unaffected despite capsaicin stimulation. Similarly, immunofluorescence analysis demonstrated the presence of PGP‐positive neurons in close proximity to K14‐positive keratinocytes of the epidermis in both unstimulated and capsaicin‐stimulated conditions (Figure 4bc,d). Immunoreactivity for junctional proteins claudin‐1 and E‐cadherin also remained unchanged following capsaicin stimulation (Figure 4be,f), suggesting barrier function was unaffected. These data demonstrate that capsaicin stimulation has had no detrimental effect on epidermal structure; a stratified, organized epidermis was conserved despite stimulation with expected expression of junctional proteins.

**FIGURE 5:**
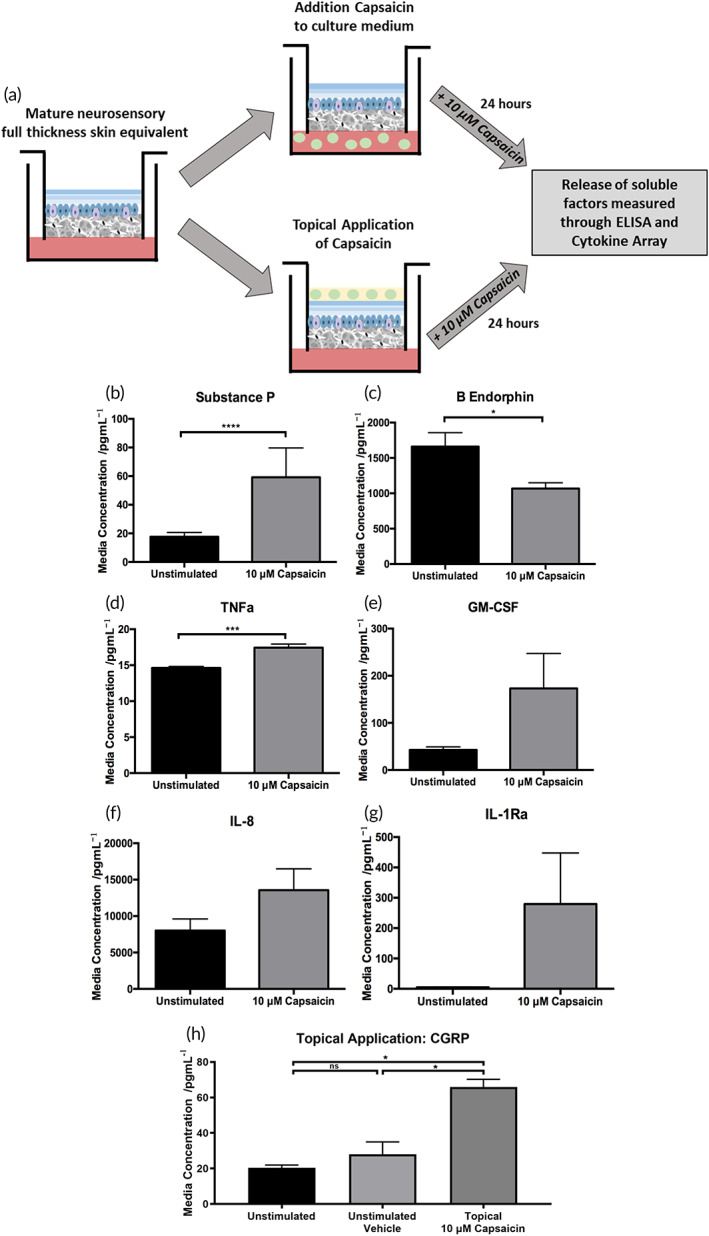
Stimulation with known sensitizing agent induces expected pro‐inflammatory response. Neurosensory FT‐HSEs were exposed to 10 μM capsaicin for 24 h in their culture medium or topically in a moisturizing formulation (a). Secretion of neuropeptide substance P is significantly increased following stimulation in the culture medium (b) as detected by ELISA. A cytokine array revealed a significant reduction in β‐endorphin (c) secretion and increased secretion of a wide range of pro‐inflammatory factors including: TNFα (d), GM‐CSF, (e) IL‐8 (f), IL‐1Ra (g). Topical application of 10 μM capsaicin induced a significant release in calcitonin gene‐related peptide (CGRP) into the culture medium (h) as detected by ELISA in a small scale pilot. Data represent mean ± SEM, *n* = 3, *p < 0.05, ***p < 0.001, ****p < 0.0001. Scale bars: 50 μm

Neuronal functionality was characterized through the secretion profiles of a wide range of signaling molecules including a neuropeptide, hormone and pro‐inflammatory cytokines, upon capsaicin stimulation. Measurements of the concentration of each signaling mediator in the culture medium of neuron‐containing FT‐HSEs were performed either by ELISA (substance P) or a commercially available array (β‐endorphin, GM‐CSF, TNFα, IL‐8, and IL‐1Ra). The concentration of substance P (Figure 5b) in the culture medium of HSEs stimulated with capsaicin was significantly increased. This is as expected, as the release of substance P from sensory neurons in response to capsaicin is a well‐documented signaling event in the transmission of pain resultant from a noxious stimulus,^64^, ^65^ demonstrating the ability of sensory‐like neurons within our FT‐HSE to respond to stimuli in a physiological manner.

The concentration of β‐endorphin (Figure 5c) in the culture medium was significantly reduced following capsaicin treatment. Granulocyte‐macrophage colony stimulating factor (GM‐CSF) has been implicated in nociceptor activation, pain signaling and neuronal sensitization,^66^, ^67^ concentration of which was also increased in the culture medium of stimulated neuron‐containing models (Figure 5e). A range of pro‐inflammatory cytokines were also found at higher concentrations in the culture medium of capsaicin stimulated FT‐HSEs, including: TNFα (Figure 5d), IL‐8 (Figure 5f), and IL‐1Ra (Figure 5g). Secretion of these cytokines is a known downstream consequence of increasing substance P‐mediated signaling in response to capsaicin stimulation.^68^ These data suggest that sensory‐like neurons are present in the dermal compartment, in close contact with keratinocytes of the epidermis and respond to a known noxious stimulus in the expected manner through release of an array of signaling molecules, thus demonstrating their functionality.

Building on this demonstration of functionality, we conducted a small pilot study, whereby 10 μM capsaicin was applied topically to the surface of innervated skin models for 24 h in a glycerin‐rich oil‐in‐water emulsion previously used as a vehicle.^3^ This resulted in a significant increase in CGRP release into the culture medium (Figure 5h) compared with both unstimulated and vehicle controls, providing proof‐of‐concept data that this model system can be applied to cosmetic and therapeutic analysis of topical preparations.

## 3 DISCUSSION

The use of HSEs for dermatological research is becoming increasingly popular due to limitations associated with animal testing and both the expense and complex nature of orchestrating clinical trials. However, many basic HSEs offer limited modeling of the downstream response to noxious stimuli due to inclusion of only two cells types: keratinocyte and fibroblasts. Although this offers a reductionist approach, their applications are often restricted due to their inability to recapitulate complex cell‐to‐cell interactions. In this study, we have developed and characterized an innervated and full‐thickness HSE, containing sensory neuron‐like cells, suitable for an array of applications.

In order to create a functional bioengineered tissue, the selection of an appropriate neuronal cell line for incorporation into an established HSE system was a crucial first step. We selected F11 hybrid neuronal cells that are a fusion product of mouse neuroblastoma cells with embryonic rat DRG cells. Due to limitations associated with the availability of human neurons, we selected this model cell line as it is also routinely used to recapitulate aspects of peripheral and sensorial neuronal networks in vitro,^69^, ^70^, ^71^, ^72^ as well as because it has been well characterized at a transcriptomic level,^73^ and known to express a range of ion channels and receptors consistent with nociceptive signaling.^74^, ^75^, ^76^ As expected upon differentiation with forskolin, cells adopted a more dendritic morphology with evidence of neurite outgrowth and positive expression for biomarkers PGP 9.5 and advillin, indicative of a sensory phenotype,^77^ thus providing a relevant source of neurons for inclusion within the HSE.

A dermal compartment was then constructed using a well‐characterized methodology involving the culture of human dermal fibroblasts within a porous polystyrene scaffold, which promotes three dimensional cell‐to‐cell interactions and secretion of endogenous ECM, providing a robust foundation for the construction of subsequent tissue layers. It is upon this dermal compartment that F11 cells were cultured and differentiated to produce an advillin and PGP 9.5 positive sensory neuron‐like cell population.

Neuron‐containing dermal compartments were found to be responsive to two stimuli: capsaicin and eugenol. Secretion of substance P, a mediator of nociceptive signaling,^78^ was used to measure neuronal responsiveness, particularly as secretion and subsequent depletion of substance P is a well‐documented consequence of capsaicin treatment.^79^, ^80^ The concentration of substance P in the culture medium when exposed to both capsaicin and eugenol increased as expected. CGRP is also a neuropeptide released from substance P‐containing nociceptive cells in response to noxious stimuli and a mediator of neuroinflammation,^81^, ^82^, ^83^ which increased in response to capsaicin and eugenol treatment.^84^ This suggests that a population of sensory‐like neurons residing on the surface of the dermal construct respond to stimuli in an expected manner.

An epidermal layer was then constructed on this established neuron‐containing dermal foundation and the resulting anatomy and neuronal localization was found to recapitulate aspects of native cutaneous neuronal location. In native human skin, cell bodies of sensory neurons are located in the DRG and nerve fibers branch within the dermis upward into the epidermis where they are exposed to external stimuli.^38^ In our bioengineered HSE, neuronal cell bodies were isolated to the upper layer of the dermal compartment with the extension of branching neurites in close proximity to basal keratinocytes of the epidermis. Re‐creating the correct tissue anatomy in correlation with the native tissue is an important step in engineering any in vitro tissue, as tissue structure and function correlate closely.^27^

To characterize the FT‐HSE, we refined our strategy and tested for sensitivity to capsaicin alone, as capsaicin‐sensitivity is specific to peripheral sensory neurons. Capsaicin treatment of neuron‐containing FT‐HSEs resulted in a significant rise in substance P secretion, the expected response to stimulation of nociceptive sensory neurons, which translates to a burning or stinging sensation in vivo.^50^, ^85^, ^86^ Similarly, secretion of pro‐inflammatory cytokines: TNFα, IL‐8, and IL‐1Ra was also increased following capsaicin treatment. This is an expected response to substance P release,^68^, ^87^ as downstream signaling results in NFκB driven transcription of inflammatory‐associated genes.^88^, ^89^

Although the neurogenic inflammatory response mediated by substance P is systemic and complex in vivo involving vasodilation, mast cell degranulation, and immune cell recruitment,^88^, ^90^, ^91^ it also known to have a direct impact on keratinocytes and fibroblasts.^89^, ^92^ We believe that this is direct evidence as to the bidirectional crosstalk between sensory‐like neurons in the dermal compartment and epidermal keratinocytes with substance P acting as a signaling intermediary, driving the pro‐inflammatory response. However, keratinocytes and fibroblasts are themselves known to express receptors responsive to capsaicin, we believe due to the significant rise in substance P detected, that the neuronal component within this model system is the driving factor responsible for pro‐inflammatory stimulation, thus capturing dynamic neuronal–keratinocyte interactions.

Similarly, GM‐CSF secretion, a cytokine known to be involved in the neuroinflammatory response through recruitment of immune cells, indirect nociception transduction, and neuronal sensitization,^66^, ^93^ was also increased following capsaicin treatment. Neuropeptide and hormone β‐endorphin was significantly reduced following capsaicin treatment. This observation is supported by previous findings in a rodent‐based model of hypothalamic capsaicin stimulation.^94^ The action of β‐endorphin in the nervous system is thought to be involved in the neurosensorial response along with pain signaling and sensitivity. β‐endorphin has been implicated in opiate signaling through binding of the μ‐opioid receptor with analgesic properties^95^ and increased plasma β‐endorphin levels have previously been linked to increased peripheral pain threshold.^96^ Therefore, this finding suggests that capsaicin stimulation in our model system is evoking a similar hormonal response to in vivo, further supporting and validating our FT‐HSE by providing evidence of functionality in‐line with expected behaviors.

In this study, we describe the successful incorporation of sensory‐like neurons into a FT‐HSE and subsequent functional response following stimulation with well‐characterized drugs. We have observed secretion of neuropeptides and downstream pro‐inflammatory cytokines typical of a neurosensorial response in vivo. However, skin neuroinflammation in vivo is a complex response that involves interaction between both the immune and nervous systems within skin tissue. Although immune cells are absent from this bioengineered tissue, advances in the field of skin tissue engineering have resulted in the generation of immunocompetent HSEs.^97^, ^98^, ^99^ Further developments in this area could conceivably combine both neuronal and immune components to produce an extremely complex bioengineered tissue, striving to recapitulate as many aspects of the native human skin response to sensorial stimulation as possible. However, this current platform offers a reductionist approach, to screen the effect of a given stimulus on the neuronal component of skin sensitization in isolation, to better understand and dissect the intricacies of cellular and molecular signaling events that arise.

While few neuron‐containing HSEs have been reported previously in the literature, they each have limitations such as poor epidermal morphology,^45^ epidermal compartment only^46^ or utilize exogenous animal‐derived ECM constituents.^47^ Here, we present a FT‐HSE system that contains a phenotypically representative subset of neurons that display characteristics of sensory neurons and their anatomically typical location, in close contact with keratinocytes of a stratified and organized epidermis. This was achieved through modification of a well‐characterized FT‐HSE system built upon a solid dermal foundation of fibroblasts and endogenous ECM components including: fibronectin, elastin, and appropriately orientated collagens.^27^, ^100^ As microenvironmental cues have a major impact on modeling tissue structure and function in vitro,^101^ an important consideration in tissue engineering any epithelial tissue is to ensure the biochemical and biophysical cues received by the cells from their surrounding ECM are physiologically relevant.

One potential disadvantage to our approach is the use of a murine hybrid neuronal cell line, as F11 cells are a fusion product of mouse neuroblastoma cells with embryonic rat DRG cells. Combining mammalian neurons with a fully humanized tissue equivalent has inherent limitations due to species specificity. However, isolation, expansion and use of human neurons in vitro has its own shortcomings, as immortalized cell lines often have limited functionality compared with their native in vivo counterpart. For this reason and to provide proof‐of‐concept data, we opted for the well‐characterized F11 cell line that is clearly documented to differentiate into sensory‐like neurons, the subtype of interest in this particular study.

Furthermore, we also demonstrate the application of such a model to screen potential cosmetics and topical preparations, an important screening step in determining the safety profile of any topically applied formulation. In this study, we applied capsaicin topically in a basic facial moisturizing formulation to the surface of innervated skin models and found a significant increase in the concentration of CGRP secreted into the culture medium. This provides proof‐of‐concept data that a functionally innervated skin model such as this can be applied to screen topical formulations befitting of both cosmetic and pharmaceutical industries.

In this study, we describe advances made to an existing platform, creating a more complex and functional FT‐HSE system that contains sensory‐like neurons and is responsive to algesic stimuli in both soluble and topical preparations. This technology provides a platform for a wide range of applications including screening of therapeutic and cosmetic treatments to determine their neurosensorial effects. The use of predictive, in vitro assays has many benefits including streamlining clinical trials and reduction in the use of animals in dermatological research.

## 4 MATERIALS AND METHODS

### 4.1 Neuronal cell culture

The F11 hybrid neuronal cell line is a fusion product of mouse neuroblastoma cells with embryonic rat DRG cells. F11 cells (Sigma‐Aldrich, Missouri, USA) were cultured in Dulbecco's Modified Eagle's Medium (DMEM, Thermo Fisher Scientific, Loughborough, UK) supplemented with 2 mM l‐glutamine (Thermo Fisher Scientific) and 10% fetal bovine serum (FBS, Thermo Fisher Scientific). Cultures were maintained in culture flasks (Greiner Bio‐One, Kremsmünster, Austria) at 37°C, 5% CO_2_ in a humidified environment, and passaged at 80% confluence at a ratio of 1:3 as per manufacturer's guidelines.

### 4.2 Generation of skin equivalents

Commercially available cells were used to create HSEs including human neonatal keratinocytes #1817888, #1944927, #2288858, and #2018512 (HEKn, Thermo Fisher Scientific), and neonatal dermal fibroblasts (HDFn) #1366356 and #1366434 (Thermo Fisher Scientific). Cells were screened for infectious agents by the manufacturer and used in HSEs within three to five passages.

Generation of HSEs was modified from a previously described methodology.^27^ HDFn were seeded onto Alvetex® Scaffold (ReproCELL Europe Ltd, Glasgow, UK) at a density of 0.2 × 10^6^ cells per scaffold and incubated with DMEM, 2 mM l‐glutamine and 10% FBS for 14 days. Following dermal maturation, F11 neuronal cells were seeded onto the scaffold and incubated in submerged culture for a further 7 days in the presence of 10 μM forskolin (Sigma‐Aldrich) to promote neuronal differentiation and neurite extension.

To form an epidermal compartment, 1.3 × 10^6^ HEKn were seeded onto each dermal compartment and cultured for 2 days in submerged culture in a proliferation promoting medium consisting of Epilife® Medium (Thermo Fisher Scientific) supplemented with human keratinocyte growth supplement (HKGS, Thermo Fisher Scientific), 10 ngml^−1^ keratinocyte growth factor (KGF, PeproTech, London, UK), 140 μM CaCl_2_ (Sigma‐Aldrich) and 10 mgml^−1^ ascorbic acid (Sigma‐Aldrich). HSEs were then raised to the ALI in high calcium conditions (1.64 mM CaCl_2_) to promote keratinocyte differentiation and epidermal stratification, and maintained for a further 14 days to form a mature epidermis for use in downstream applications.

### 4.3 Skin equivalent stimulation

Neuron‐containing HSEs were stimulated with known neuroactive compounds either 10 μM capsaicin (Sigma‐Aldrich) or 100 μM eugenol (Sigma‐Aldrich). Compounds were added to the culture medium either in submerged cultured (dermal equivalent) or at ALI (FT‐HSE) for 24 h. For topical application, 10 μM capsaicin was added to a glycerin‐rich oil‐in‐water emulsion (as previously described in the literature^3^) and mixed. 2 μl of capsaicin containing formulation was added using a positive displacement pipette to the surface of a mature neuron‐containing HSE and spread evenly across the surface using a glass rod. After 24 h treatment, samples were harvested for analysis.

### 4.4 ELISA culture medium analysis

Culture medium was harvested from bioengineered skin cultures containing human sensory neurons (both dermal equivalents and FT‐HSEs) either unstimulated or stimulated with 10 μM capsaicin or 100 μM eugenol for 24 h. Commercially available ELISA kits were used to determine the concentration of substance P (R&D Systems, Minneapolis, USA, #KGE007) or CGRP (Abexxa, Cambridge, UK abx574133) in the culture medium and manufacturer's instructions were followed.

### 4.5 Cytokine array

Samples of culture medium were harvested from neuron‐containing FT‐HSEs either unstimulated or stimulated with 10 μM capsaicin for 24 h. Analysis of the cytokine content of the medium was performed by Eve Technologies (Calgary, Canada) and two arrays were conducted: Human Cytokine Proinflammatory Focused 15‐Plex Discovery Assay® Array (HDF15) and Rat/Mouse Neuropeptide 5‐Plex Assay for Tissue/Cell Culture samples (RMNP‐05‐200).

### 4.6 Paraffin wax embedding

Samples were fixed in 4% paraformaldehyde (Sigma‐Aldrich) overnight at 4°C and then dehydrated through a series of ethanols. Samples were incubated in Histoclear (Scientific Laboratory Supplies, Nottingham, UK) alone, then mixed 50:50 with molten paraffin wax (Thermo Fisher Scientific) followed by paraffin wax alone. Samples were embedded in plastic molds (CellPath, Newton, UK) with paraffin wax and sectioned transversely using a microtome (Leica RM2125RT). The 5 μm sections were captured onto charged microscope slides (Thermo Fisher Scientific).

### 4.7 Hematoxylin and eosin staining

Samples were deparaffinized in Histoclear (Scientific Laboratory Supplies) and rehydrated ethanols. Samples were then incubated in Mayer's hematoxylin (Sigma‐Aldrich) for 5 min followed by alkaline ethanol for 30 s to blue the nuclei. Slides were dehydrated through a series of ethanols, incubated with eosin (Sigma‐Aldrich) for 30 s and further dehydrated in ethanol. Finally, slides were cleared in Histoclear and mounted with coverslips using Omnimount mountant (Scientific Laboratory Supplies).

### 4.8 Immunofluorescence

Sections were deparaffinized in Histoclear and rehydrated through a series of ethanols. Antigen retrieval was performed in citrate buffer pH 6 (Sigma‐Aldrich) at 95°C for 20 min, followed by blocking and permeabilization for 1 h with a solution containing: 20% neonatal calf serum (NCS, Sigma‐Aldrich) and 0.4% Triton X‐100 (Sigma‐Aldrich) in phosphate buffered saline (PBS). Samples were then incubated overnight at 4°C in primary antibody diluted in blocking buffer (α‐SMA, Abcam, Cambridge, UK, ab7817, 1:100), (Advillin, Thermo Fisher Scientific, # BS‐11451R, 1:100), (Claudin‐1, Abcam, ab15098, 1:250), (Collagen 1, Abcam, ab34710, 1:100), (Collagen 3, Abcam, ab7778, 1:100) (E‐cadehrin, BD Biosciences, Berkshire, UK, 610182, 1:100), (PGP 9.5, Abcam, ab108986, 1:100), (Keratin‐14, Abcam, ab7800, 1:100), (TUJ‐1, Cambridge Bioscience, Cambridge, UK, 3525‐100, 1:600), (Vimentin, Abcam, ab92547, 1:100). Slides were washed three times in PBS and incubated with the appropriate secondary antibody diluted in blocking buffer for 1 h at room temperature (donkey anti‐rabbit Alexa® Fluor 488 or donkey anti‐mouse Alexa® Fluor 594, Thermo Fisher Scientific, 1:1000) and washed three times in PBS. Finally, slides were mounted using Vectashield Hardset with DAPI (Vector Laboratories, Peterborough, UK).

### 4.9 Light microscopy

Histology images were captured using Leica ICC50 high‐definition camera and Brightfield microscope. Immunofluorescence images were taken using the Zeiss 880 confocal microscope with Airyscan and Zen software.

### 4.10 Epidermal thickness measurement

Epidermal thickness was measured using Image J software as previously described.^3^ Briefly the software scale was calibrated and the straight line tool was used to take measurements from the basement membrane to the top of the viable epidermis, excluding *stratum corneum* layers. Ten measurements were taken per image and three images analyzed per skin model.

### 4.11 Statistical analysis

GraphPad Prism software was used to measure the statistical significance by use of a Student's T‐test or one‐way ANOVA as appropriate. *p ≤ 0.05, **p ≤ 0.01, ***p ≤ 0.001, ****p ≤ 0.0001.

## 5 CONCLUSIONS

In this study, we have described the development of a novel, complex, innervated HSE that is able to respond to neurosensitizing stimuli in an anticipated manner and in‐line with the known physiological response. This demonstrates the potential application of a complex, multicellular, bioengineered tissue, to be used as a predictive tool in clinical research to screen both individual actives and complex topical formulations and any subsequent neuron‐mediated inflammatory response within the skin. Ultimately of benefit to cosmetic, therapeutic or academic pursuits, through providing a robust, reproducible and responsive platform technology. However, building upon this pioneering study providing proof‐of‐concept data as to the inclusion of neurons into a full‐thickness skin equivalent system, future work will aim to incorporate human‐sourced neurons, particularly as stem cell technology advances.

## AUTHOR CONTRIBUTIONS

**Matthew Freer:** Data curation (lead); formal analysis (lead); investigation (lead); methodology (lead); validation (lead); writing – review and editing (equal). **Nicole Darling:** Data curation (equal); formal analysis (equal); investigation (equal); writing – review and editing (equal). **Kirsty Goncalves:** Writing – original draft (lead). **Kevin Mills:** Conceptualization (lead); funding acquisition (lead); project administration (equal); supervision (equal); writing – review and editing (equal). **Stefan Przyborski:** Conceptualization (lead); project administration (lead); resources (lead); supervision (lead); visualization (equal); writing – review and editing (equal).

## CONFLICT OF INTEREST

Kevin J. Mills is a full‐time employee of Procter & Gamble (Cincinnati, OH, USA). This work was supported by funding from Procter & Gamble. All other authors declare no conflicts of interest.

### PEER REVIEW

The peer review history for this article is available at https://publons.com/publon/10.1002/btm2.10484.

## Data Availability

No datasets were generated or analyzed during the current study.
